# Ovarian follicular dynamics during the estrous cycle in locally adapted Curraleiro Pé-Duro cows

**DOI:** 10.1590/1984-3143-AR2025-0019

**Published:** 2025-10-27

**Authors:** Rodrigo Dorneles Tortorella, Isabela Maria Lopes, Joseane Padilha da Silva, Jairo Pereira Neves, Alexandre Floriani Ramos

**Affiliations:** 1 Faculdade de Agronomia e Medicina Veterinária, Universidade de Brasília – UnB, Brasília, DF, Brasil; 2 Embrapa Recursos Genéticos e Biotecnologia, Brasília, DF, Brasil; 3 Universidade Estadual de Londrina – UEL, Londrina, PR, Brasil; 4 Universidade Professor Edson Antônio Velano, Alfenas, MG, Brasil

**Keywords:** animal genetic resources, bovine, estrous cycle, follicular waves, naturalized breeds

## Abstract

This study aimed to characterize the ovarian follicular dynamics in locally adapted Curraleiro Pé-Duro cows and heifers. Cyclic heifers (n =12) and non-lactating, multiparous cows (n = 11) were examined daily by ultrasonography for two consecutive ovulations (an estrous cycle). Follicles > 3 mm and corpus luteum (CL) were measured and followed until they disappeared. Follicular and luteal characteristics were not different between heifers and cows. Consequently, data on cows and heifers were combined according to the number of follicular waves. Follicular dynamics was characterized by the predominance of two (36.8%) and three (63.2%) follicular waves. No difference in estrous cycle length between these follicular wave patterns was observed. The number of recruited follicles was smaller in the second follicular wave. The ovulatory follicle (OF) growth rate (mm/d) and maximum diameter were greater (P < 0.05) in females, showing three waves. The ovulatory wave was shorter (P < 0.05) than the preceding waves regardless of the wave pattern. No difference was found in CL development between females with two and three follicular wave patterns. Some follicular dynamics characteristics were similar to *Bos taurus* and others similar to *Bos indicus*, confirming the crosses made throughout the years. The data from this study will be useful to better estrous cycle manipulation aiming for good results in artificial insemination (AI), fixed-time AI (FTAI), and multiple ovulation and embryo transfer (MOET) programs.

## Introduction

The demand for quality meat is likely to increase due to population growth, and the economic scenario indicates that consumers will be increasingly demanding regarding the origin of their food and the use of chemical products such as antibiotics and antiparasitics. There is a prospect of increased global climate change, with reduced rainfall and rising temperatures, thus affecting cattle production systems and increasing the need to use genotypes that are better adapted to hostile conditions. In this context, the use of “locally adapted” animals can be an alternative for meat production with low investment and high added value (Neiva et al., 2011).

Locally adapted breeds, such as Curraleiro Pé-Duro, originate from cattle brought by Portuguese and Spanish colonizers to Latin America over 500 years ago. In Brazil, its rearing was established in areas with low pasture quality, humidity, and high temperatures (Northeast and Midwest), resulting in rustic animals that are resistant to unfavorable conditions (Vieira et al., 2022). This rusticity is characterized by greater resistance to diseases in general, ectoparasites, endoparasites, and poisoning (Serodio et al., 2019). However, its population suffered a significant decline due to the intense genetic selection pressure carried out on the exotic breeds *Bos taurus* and *Bos indicus* and their disordered crossings with Curraleiro Pé-Duro (Fioravanti et al., 2011).

The preservation and expansion of the breed require the use of reproductive biotechnologies such as fixed-time artificial insemination (FTAI) and multiple ovulation and embryo transfer (MOET), for example. Knowledge of the breed’s reproductive physiology is necessary for successful FTAI and/or MOET (Tortorella et al., 2016, 2017; Teixeira et al., 2017; Santos et al., 2018), which is not yet fully understood for Curraleiro Pé-Duro. Several studies on follicular dynamics have been conducted in the *Bos taurus* (Ginther et al., 1989; Sartori et al., 2001; Bonato et al., 2022) and *Bos indicus* (Figueiredo et al., 1997; Morotti et al., 2018; Alvarez et al., 2023) breeds genetically selected by humans, leading to good hormonal manipulation and increased reproductive indices (Alves et al., 2021; Consentini et al., 2021). However, there are physiological differences between these two bloodlines (Baruselli et al., 2007), leading to modifications in hormonal manipulation depending on the worked group. Therefore, knowing the possible reproductive particularities of Curraleiro Pé-Duro is important for better control of the estrous cycle and increased reproductive efficiency. This study aimed to characterize and compare the follicular dynamics of Curraleiro Pé-Duro heifers and cows raised in a dry tropical climate environment.

## Methods

### Location, feed management, and animals

All procedures described herein were approved (approval number: 02/2013) by the Animal Health and Welfare Committee at Embrapa Cenargen, Brasília, Federal District, Brazil.

The experiments were conducted at a ranch located in Brasília, DF, Brazil (15°52′-15°56′S and 48°00′-48°02′W), with altitudes ranging from 1050 to 1250 m. The climate is the Koppen Aw, indicating a dry winter (relative humidity can be as low as 10%) and a rainy summer.

Eleven multiparous cows ranging from 3 to 8 years old, and twelve Curraleiro Pé-Duro heifers from 2 to 3 years old with a mean body condition score (BCS) of 3 on a scale from 1 to 5 (where 1 = emaciated and 5 = obese; Wildman et al., 1982) were used in this study. Multiparous cows and heifers had a mean live weight of 320 kg and 260 kg, respectively. During the experimental period, the animals were maintained by continuous grazing of *Brachiaria decumbens* with mineralized salt and water provided ad libitum.

### Estrus synchronization and ultrasonographic examination

The cows were evaluated by rectal palpation, ultrasonography (Mindray 2200 Vet, Shenzhen, China, equipped with a 7.5-MHz transrectal transducer), and vaginoscopy before the beginning of the Experiment to determine the absence of diseases or abnormalities in their reproductive tract.

The estrous cycles of nonlactating Pé-Duro females (n = 23) were synchronized with two injections of D-Cloprostenol i.m. (0.150 mg PGF2α; Prolise^®^, Tecnopec LTDA, São Paulo, Brazil) administered 11 days apart. After the second PGF2α injection, the cows were observed for half an hour twice daily for estrous behavior with the aid of a vasectomized bull. The onset of estrus was considered to have occurred when a cow stood to be mounted by another cow or vasectomized bull.

Ultrasonographic examinations were performed daily (08:00 to 10:00 am) during one complete estrous cycle, from the day next to the last injection of PGF2α to Day 2 of the subsequent cycle. The duration of the estrous cycle was characterized by the period (days) between two ovulations. During each examination, ovarian maps were drawn to record the diameter and relative position of follicles ≥ 3 mm and CL. The follicular diameter was determined by the average of two measured diameters of each follicle. Ovulation (Day 0) was defined as the disappearance of a previously identified dominant follicle (DF, ≥ 10 mm) from one ultrasound examination to the next and was confirmed by subsequent detection of a corpus luteum (CL). The CL volume was assessed at 5, 10, and 15 post-ovulation days using the formula V = 4/3πR^3^, where R = (Da/2 + Db/2)/2 and Da and Db were the perpendicular diameters of CL. A CL with a cavity was calculated using the same formula and deleted from the total volume of CL. The lifespan of CL was characterized from the first identification to the last one before the next ovulation.

A follicular wave was characterized by the initial growth of a group of follicles around 4 mm and, subsequently, the formation of a DF and a group of smaller follicles (subordinate follicles, SF). The day of emergence was defined as the last day DF was 4 mm in a retrospective analysis. DF was identified as the largest follicle that grew to a diameter of ≥ 10 mm and was at least 2 mm larger than SF. The last DF in the estrous cycle was considered the ovulatory follicle (OF). The lifespan of DF and the largest SF were characterized by the days between follicle emergence and its complete atresia or ovulation for OF. A growing and static phase characterized the dominance (days) of DF. The growth rate (mm/d) of DF and the largest SF were determined from the day the follicle was first identified to the day the diameter no longer increased or ovulated for OF.

### Statistical analyses

The statistical analysis was carried out using R Core Team 2013 free software (R: A language and environment for statistical computing. R Foundation for Statistical Computing, Vienna, Austria) and significance was considered for P < 0.05.

Initially, the statistical model had the animal category (cow vs. heifer) as the main group effect. However, a great similarity between the groups after comparing the means of the follicular characteristics using the Bootstrap method. Therefore, the database was combined, and the mean effect of the follicular characteristics was compared according to the number of waves (2 vs. 3). The adjustments of the follicular and luteal development curves were performed using the linear regression model (least squares method), with the response variable consisting of the size of DF and CL, respectively, and the covariate consisting of the days of evaluation. The coefficient of determination (R^2^) and the graphical analysis of residuals were used as criteria to assess the quality of the curve adjustment. The comparison of means between the groups with 2 and 3 waves for the various follicular and luteal characteristics was performed using the Bootstrap method. This method was chosen because the available sample size was not large enough to guarantee the necessary assumptions for applying the classical tests of comparison of means. The data were presented as mean ± standard error of the mean (SEM).

## Results

One cow and one heifer were excluded from the analysis because they did not have normal cyclicity (estrous cycle length from 18 to 23 days). One heifer was excluded because she had four follicular waves, and another one was excluded because the rectum was too sensible, making the exam difficult to perform.

**Figure 1:**
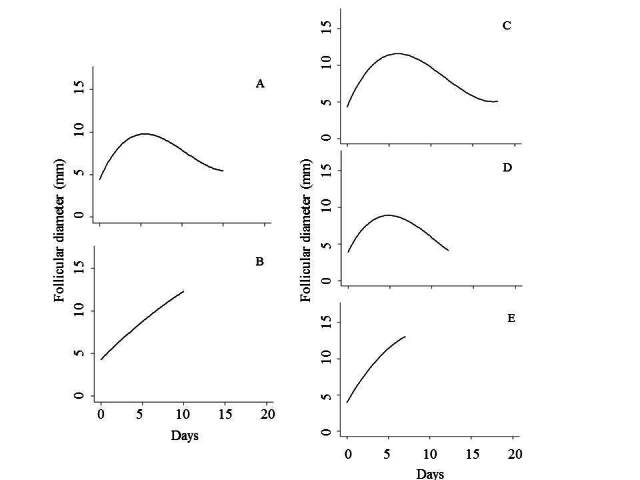
Regression equation of the first and second DF in females with two follicular waves (A and B, respectively) and the first, second, and third DF in females with three follicular waves (C, D, and E, respectively). A = 4.383041 + 2.241644 x – 0.277616 x^2^ + 0.0088 x^3^, R^2^ = 0.51; B = 4.27146 + 0.98906 x – 0.01846 x^2^, R^2^ = 0.89; C = 4.325581 + 2.731449 x – 0.305909 x^2^ + 0.008688 x^3^, R^2^ = 0.58; D = 3.87059 + 2.31368 x – 0.31202 x^2^ + 0.01007 x^3^, R^2^ = 0.67; E = 3.95774 + 1.98322 x – 0.09814 x^2^, R^2^ = 0.91. y = follicular diameter and x = assessment day. Follicular waves were normalized to day 0 whether it was the first, second, or third.

Heifers (n = 9) and cows (n = 10) displayed basically two types of follicular waves, two (33.33%, n = 3; 40%, n = 4) and three (66.67%, n = 6; 60%, *n* = 6) wave patterns, respectively. The studied follicular characteristics were not different (P > 0.05) for cows and heifers with the same number of follicular waves. Consequently, data from cows and heifers were combined, and follicular and luteal characteristics were compared between two and three-wave animals (Figure 1 and Table 1).

**Table 1:** Comparison of follicular characteristics and dominant follicles (DF; mean ± SEM) between Curraleiro Pé-Duro females with two or three follicular waves.

**Parameters**	**Follicular Waves**	
	**Two (n = 7)**	**Three (n = 12)**
Interovulatory interval (days)	21.4 ± 0.4	22.3 ± 0.6
Interval from onset of estrus and ovulation (hours)	24.8 ± 3.0	26 ± 2.0
Day at emergence of the 1st follicular wave*	0.4 ± 0.3	0.2 ± 0.1
Day at emergence of the 2nd follicular wave*	11.1^a^ ± 0.4	9.1^b^ ± 0.6
Day at emergence of the 3rd follicular wave*	-	15.1 ± 0.6
Number of recruited follicles in the 1st wave	10.8 ± 1.3	12.3^A^ ± 0.9
Number of recruited follicles in the 2nd wave	10 ± 1.8	8.4^B^ ± 0.9
Number of recruited follicles in the 3rd wave	-	14.1^A^ ± 1.3
Life-span of the 1st DF (days)	14.2^A^ ± 1.1	15.3^A^ ± 0.8
Life-span of the 2nd DF (days)	10^b,B,1^ ± 0.3	11.4^a,B^ ± 0.5
Life-span of the 3rd DF (days)	-	7.2^C,2^ ± 0.2
Dominance phase of the 1st DF (days)	8.7 ± 0.6	9.3^A^ ± 0.5
Dominance phase of the 2nd DF (days)	10^a,1^ ± 0.3	7^b,B^ ± 0.5
Dominance phase of the 3rd DF (days)	-	7.2^B,2^ ± 0.2
Regression phase of the 1st DF (days)	5.5 ± 0.64	5.9 ± 0.5
Regression phase of the 2nd DF (days)	-	4.5 ± 0.4
Growth rate of the 1st DF (mm/d)	1 ± 0.1	1^B^ ± 0.1
Growth rate of the 2nd DF (mm/d)	0.8^2^ ± 0.1	0.9^B^ ± 0.1
Growth rate of the 3rd DF (mm/d)	-	1.2^A,1^ ± 0.1
Maximum diameter of the 1st DF (mm)	10.6^b,A^ ± 0.3	12.5^a,A^ ± 0,4
Maximum diameter of the 2nd DF (mm)	12,1^a,B,2^ ± 0,2	9.9^b,B^ ± 0.2
Maximum diameter of the 3rd DF (mm)	-	13.1^A,1^ ± 0.3
Day at maximum diameter of the 1st DF*	5.8 ± 0.9	6.5 ± 0.5
Day at maximum diameter of the 2nd DF*	20^a^ ± 0.5	14.4^b^ ± 0.8
Day at maximum diameter of the 3rd DF*	-	21.5 ± 0.6

^a,b^ Means with different superscripts within a row differed (*P* < 0.05). ^A,B,C^ Means with different superscripts within a column differed (*P* < 0.05). ^1,2^Ovulatory follicles (last DF of the wave) means with different superscripts differed (*P* < 0.05). *Day 0: day of ovulation.

### Follicular dynamics

Follicular dynamics in Curraleiro Pé-Duro females was characterized by two (2W, 36.8%; n = 7) and three (3W, 63.2%; n = 12) follicular waves (Table 1).

Females with 2W and 3W differed (*P* < 0.05). In 2W females the emergence of the 2nd follicular wave started earlier, and the maximum diameter of the 2nd DF was larger. In 3W females the lifespan of the 2nd DF was shorter; the lifespan of the OF was shorter; the dominance phase of the 2nd DF was shorter; the dominance phase of the 2nd DF was shorter; the dominance phase of the OF was shorter; the maximum diameter of the 1st DF was larger; the maximum diameter of the OF was larger and the day at maximum diameter of the 2nd DF was earlier.

Some parameters within the group of females in 2W differed, as follows: the lifespan of the 2nd DF was shorter than the 1st DF, and the maximum diameter of the 2nd DF was larger than the 1st DF.

Moreover, some parameters regarding females in 3W differed, as follows: the number of recruited follicles in the 2nd wave was smaller than in the 1st and 3rd wave; the lifespan of the 1st DF was longer than the 2nd and 3rd DF and, further, the lifespan of the 2nd DF was longer than the 3rd DF; the dominance phase of the 1st DF was longer than the 2nd and 3rd DF; the maximum diameter of the 2nd DF was smaller than the 1st and 3rd DF; and the growth rate of the 3rd DF was higher than the 1st and 2nd DF.

### CL volume (mm^3^)

**Figure 2:**
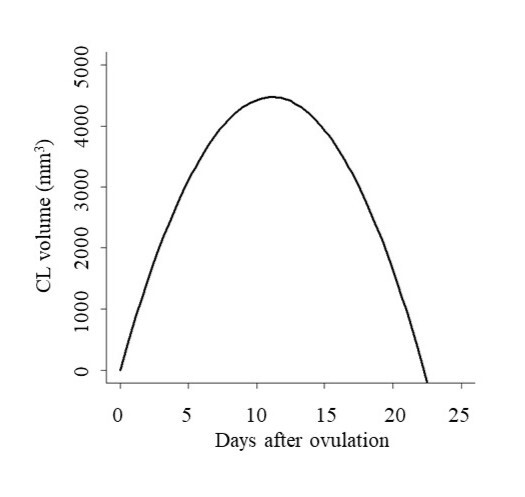
Regression equation of CL volume (mm^3^) in Curraleiro Pé-Duro females (*n* = 19). y = 787.66 x – 34.44 x^2^, R^2^ = 0.93. Y = CL volume and x = assessment day.

Females with 2W and 3W were not different (P > 0.05) for luteal characteristics. Consequently, the data from 2W and 3W females were combined (Table 2 and Figure 2).

**Table 2:** Characteristics of CL growth development (mean ± SEM) in Curraleiro Pé-Duro females.

**Parameters**	**Mean values (n = 19)**
Life-span of the CL (days)	20.6 ± 1.8
Growth phase (days)	7.7 ± 2.1
Static phase (days)	6.2 ± 2.4
Regression phase (days)	6.7 ± 1.8
Maximum volume (mm^3^)	5,486 ± 1,656
Day at maximum volume*	9.4 ± 2.5

* Day 0: day of ovulation.

CL lifespan was 20.6 ± 1.8 days with the growth and maintenance phase of luteal volume lasting 7.7 ± 2.1 days and 6.2 ± 2.4 days, respectively. After this the morphological regression lasted for 6.7 ± 1.8 days. The maximum luteal volume (5,486 ± 1,656 mm^3^) was reached 9.4 ± 2.5 days after ovulation.

## Discussion

To our knowledge, this is the first complete description of the period between two ovulations in females of the locally adapted Curraleiro Pé-Duro breed. The results demonstrated no difference in follicular and luteal development between cows (multiparous and non-lactating) and heifers. Alvarez et al. (2023) found that the pattern of follicular growth and luteal function appear to be unaffected over the years despite the increase in ovarian size and lower follicular population.

There was a predominance of two patterns of follicular development, 2W (36.8% = 7/19) and 3W (63.2% = 12/19), corroborating other studies (Alvarez et al., 2023; Baldrighi et al., 2022). The occurrence of only one female with 4 follicular waves in the present study agrees with data in the literature (Figueiredo et al., 1997; Satheshkumar et al., 2015; Ramesh et al., 2022), in which this wave pattern was rarely observed. Some studies have reported a higher occurrence of animals with 3 follicular waves (Savio et al., 1988; Satheshkumar et al., 2015), while others have found a higher proportion of animals with two waves (Townson et al., 2002; Wolfenson et al., 2004; Hassan et al., 2021), thus explaining this divergence. The difference in results may originate from genetic predisposition, different environmental conditions, and nutritional factors (Satheshkumar et al., 2015; Alvarez et al., 2023). These factors can influence serum estradiol-17β (E_2_) levels and, consequently, the number of follicle-stimulating hormone (FSH) peaks that stimulate the initiation of a new follicular wave (Adams et al., 1992; Tortorella et al., 2017).

The interval between ovulations was similar between 2W (21.4 ± 0.4 d) and 3W (22.3 ± 0.6 d), which is contrary to what has been observed in other studies (Wolfenson et al., 2004; Jaiswal et al., 2009), in which animals with three follicular waves had a longer interval between ovulations. However, Baldrighi et al. (2022) and Alvarez et al. (2023) also found no difference in the interval between ovulations in animals with two and three follicular waves in *Bos taurus* heifers and cows.

Ginther et al. (1989) reported that the appearance of the third follicular wave would be related to the longer duration of the luteal phase, which was not observed in the present study (19.8 ± 0.7 d, 2W vs 21 ± 0.5 d, 3W) or other studies such as that by Abdelnaby et al. (2018). The hypothesis that the duration (days) of the first follicular wave would determine the wave pattern (Jaiswal et al., 2009) was also not observed in the study given the equality in duration (14.2 ± 1.0 d, 2W vs 15.3 ± 0.7 d, 3W) and dominance phase (8.7 ± 0.6 d, 2W vs 9.3 ± 0.4 d, 3W) of DF. Noseir (2003) attributed the emergence of the third wave to the fact that the second DF was not able to reach a diameter and serum E_2_ concentration higher than 10 mm and 5 pg/ml, respectively, following the follicular size observed in the study.

Regardless of the group (2W or 3W), the moment of follicular emergence of the first wave was verified within 24 hours after ovulation, being earlier than that reported for *Bos indicus* females (Figueiredo et al., 1997), but similar to *Bos taurus* (Ginther et al., 1989) and Curraleiro Pé-Duro (Tortorella et al., 2017) females evaluated in another experiment. The day of emergence of the second wave (11.1 ± 0.4 d) for 2W animals and the second (9.1 ± 0.6 d) and third waves (15.1 ± 0.6 d) for 3W animals corroborates other studies using *Bos taurus* and *Bos indicus* animals (Ahmad et al., 1997; Satheshkumar et al., 2015; Baldrighi et al., 2022). Jaiswal et al. (2009) suggested that the early emergence of the second wave in animals with three waves could be related to the shorter period of dominance of the 1st DF. However, no difference was observed in the present study regarding the dominance phase of the 1st DF (8.7 ± 0.6 d, 2W vs 9.3 ± 0.4 d, 3W), as in other studies (Alvarez et al., 2023; Noseir, 2003).

The number of follicles at the beginning of the follicular waves was similar to that found by Savio et al. (1988), Abdelnaby et al. (2018) and Ramesh et al. (2022) in heifers and Holstein cows and Mithun cows (*Bos frontalis)*, respectively, but lower than that observed by Alvarez et al. (2000) in lactating multiparous cows of the Angus, Brahman, and Senepol breeds. The differences may be associated with different hormonal concentrations of E_2_, FSH, insulin-like growth factor (IGF-1), insulin, and growth hormone (GH) between *Bos taurus* and *Bos indicus* breeds (Alvarez et al., 2000; Baldrighi et al., 2022).

The duration and dominance phase (7.2 ± 0.2 d) of OF in 3W were shorter than in the previous dominants but the growth rate (1.2 ± 0.1 mm/d vs 0.9 ± 0.1 mm/d and 1 ± 0.1 mm/d) was higher in OF. The duration of OF (10 ± 0.3 d) in 2W was shorter than that of the 1st DF (14.2 ± 1.1 d) but the dominance phase (10 ± 0.3, 1st DF, and 8.71 ± 0.6 d, OF) and growth rate (0.80 ± 0.03 mm/d, 1st FD, and 1.02 ± 0.09 mm/d, FO) were similar for both dominant follicles, unlike animals with 3W. The comparison of OF of 2W vs 3W animals shows that the former had a longer duration and dominance phase than the latter, corroborating other studies (Townson et al., 2002; Baldrighi et al., 2022; Alvarez et al., 2023). Mihm et al. (2006) reported that longer follicle duration contributes to aging and lower quality of the ovulated oocyte, largely due to greater exposure to high concentrations of E_2_ and LH before ovulation.

In addition to the longer duration of OF in animals with 2W, the growth rate (0.8 ± 0.1 mm/d) was lower than that of those with 3W (1.2 ± 0.1 mm/d), corroborating the data by Ramesh et al. (2022), who observed 0.87 ± 0.01 mm/d and 1.09 ± 0.08 mm/day, respectively, for animals with two and three waves. The higher growth rate of OF in 3W animals was probably due to the lower P_4_ concentration observed during much of the development of the third wave, especially after luteolysis (Abdelnaby et al., 2018; Alvarez et al., 2023). The lower P_4_ concentration together with the elevation of the hormone estradiol 17-β allows a greater pulsatility of the luteinizing hormone (LH), thus stimulating follicular growth (Alvarez et al., 2000). Several studies have related the larger size of OF with a higher ovulation rate, luteal volume, P_4_ concentration, and pregnancy rate (Sartori et al., 2001; Tortorella et al., 2013; Alves et al., 2021; Bonato et al., 2022).

The DF size in animals with 2W (10.6 ± 0.3 mm, 1st DF and 12.1 ± 0.2 mm, 2nd DF) and 3W (12.5 ± 0.4 mm, 1st DF, 9.9 ± 0.2 mm, 2nd DF, and 13.1 ± 0.3 mm, 3rd DF) was similar to that found in *Bos indicus* females (10 to 12 mm, Alvarez et al., 2023; and 13 to 14 mm, Morotti et al., 2018) and in other studies in Curraleiro Pe-Duro females (12 to 13 mm, Tortorella et al., 2016; 11 to 12 mm, Santos et al., 2018). However, studies in *Bos taurus* animals have reported a larger DF size than that observed here (13 to 15 mm, Jaiswal et al., 2009; 15 to 16 mm, Baldrighi et al., 2022; and 15 to 16 mm, Bonato et al., 2022). The larger DF in *Bos taurus* breeds may be related to lower plasma concentrations of insulin (Baldrighi et al., 2022) and IGF-1 (Alvarez et al., 2000), leading to the need for higher follicular development to produce satisfactory concentrations of estradiol 17-β that stimulate adequate LH pulsatility and, consequently, ovulation. Another possible reason for the OF size being similar to that of *Bos indicus* animals may be associated with the higher resistance and rusticity of the Curraleiro Pé-Duro breed (Serodio et al., 2019; Vieira et al., 2022). According to Al-Katanani et al. (2002), cows that have an alteration in energy metabolism to maintain body temperature, for example, have lower aromatase enzyme activity in granulosa cells and delayed follicular development. In this case, *Bos taurus* animals would be more susceptible to this alteration and, consequently, would need more time for the follicle to reach ovulatory capacity, leading to larger OF dimensions.

The first visualization of CL occurred 2 days after ovulation in all animals, and its monitoring could be carried out until approximately 1 day before the next ovulation. CL grew around 7-9 days and remained stable until approximately 14 days after its visualization (±16 days of the cycle), when morphological regression began, corroborating the data by Hassan et al. (2021) and Baldrighi et al. (2022). The maximum luteal volume (5,486 ± 1,656 mm^3^) reached around 9-11 days after ovulation agrees with that found by Wolfenson et al. (2004) and Teixeira et al. (2017) in the Hereford and Curraleiro Pé-Duro breeds, respectively. However, Figueiredo et al. (1997) and Morotti et al. (2018) worked with *Bos indicus* animals and found a smaller luteal size than that observed in the present study. Tortorella et al. (2013) and Bonato et al. (2022) found that a larger OF gives rise to a larger CL, which was not observed in 3W animals relative to 2W. Variations in the methodology of luteal monitoring, animal category, time of work, and nutritional intake may be involved in the differences between one study and another.

The discrepancies in follicular dynamics between Curraleiro Pe-Duro and *Bos taurus* and *Bos indicus* animals may be a consequence of the crossbreeding carried out in Curraleiro Pe-Duro with Zebu cattle over the years (Santos et al., 2005).

## Conclusion

The study demonstrated that there are no differences in follicular dynamics between cyclic and non-lactating Curraleiro Pé-Duro heifers and cows, with a predominance of the 3-wave follicular pattern. Curraleiro Pé-Duro has some follicular characteristics similar to *Bos taurus* animals and others similar to *Bos indicus*, corroborating the idea that planned or unplanned crosses have been performed over the years. The acquired knowledge will serve as a basis for the development of hormonal protocols, which aim to manipulate the estrous cycle for the use of AI, FTAI, and MOET to increase the number of descendants and stimulate breeding.

## Data Availability

Research data is available in a repository.
